# Changes in hyaluronan deposition in the rat myenteric plexus after experimentally-induced colitis

**DOI:** 10.1038/s41598-017-18020-7

**Published:** 2017-12-15

**Authors:** Viviana Filpa, Michela Bistoletti, Ilaria Caon, Elisabetta Moro, Annalisa Grimaldi, Paola Moretto, Andreina Baj, Maria Cecilia Giron, Evgenia Karousou, Manuela Viola, Francesca Crema, Gianmario Frigo, Alberto Passi, Cristina Giaroni, Davide Vigetti

**Affiliations:** 1 Department of Medicine and Surgery, University of Insubria, via H. Dunant 5, Varese, Italy; 2 Department of Internal Medicine and Therapeutics, Section of Pharmacology, University of Pavia, Pavia, Italy; 3 Department of Biotechnology and Life Sciences, University of Insubria, Varese, Italy; 4 Department of Pharmaceutical and Pharmacological Sciences, University of Padova, Padova, Italy

## Abstract

Myenteric plexus alterations hamper gastrointestinal motor function during intestinal inflammation. Hyaluronan (HA), an extracellular matrix glycosaminoglycan involved in inflammatory responses, may play a role in this process. In the colon of control rats, HA-binding protein (HABP), was detected in myenteric neuron soma, perineuronal space and ganglia surfaces. Prominent hyaluronan synthase 2 (HAS2) staining was found in myenteric neuron cytoplasm, suggesting that myenteric neurons produce HA. In the myenteric plexus of rats with 2, 4-dinitrobenzene sulfonic (DNBS)-induced colitis HABP staining was altered in the perineuronal space, while both HABP staining and HA levels increased in the *muscularis propria*. HAS2 immunopositive myenteric neurons and HAS2 mRNA and protein levels also increased. Overall, these observations suggest that inflammation alters HA distribution and levels in the gut neuromuscular compartment. Such changes may contribute to alterations in the myenteric plexus.

## Introduction

A complex interplay of immunological, genetic and environmental factors is suggested to contribute to the pathogenesis of inflammatory bowel diseases (IBD), whose symptoms include disturbed sensory, secretory and motor gastrointestinal functions^1^. Mucosal damage, abnormal secretion and visceral sensation may represent early transient symptoms following the inflammatory insult to the gut, which may lead to more persistent alterations of the smooth muscle layer and enteric neuronal circuitries, resulting in dismotility. In the myenteric plexus, reduction of total neuron number, changes in chemical coding, nerve bundle and ganglia hypertrophy and/or nerve bundle hyperplasia, may contribute to alter intestinal motility patterns^2^. However, the mechanism/s underlying derangement of myenteric neuronal circuitries during gut inflammation have not been completely unraveled yet. In this context, it is particularly important to unveil possible modulators of pro-inflammatory states, in order to prevent the incidence of more evident inflammatory conditions. Besides soluble mediators (e.g. neurotransmitters, hormones, growth factors), extracellular matrix molecules provide an important framework for the enteric microenvironment and may influence the homeostasis of myenteric neuronal circuitries during pathological conditions, including IBD^3,4^. Hyaluronan (HA), an unbranched glycosaminoglycan (GAG) component of the extracellular matrix (ECM), may participate to both acute and chronic inflammatory responses in the gut^5^. HA is produced by a family of three transmembrane synthases (HAS1, HAS2, HAS3), all of which have been shown to synthesize HA in neurons, although with different molecular weights and at different speed depending on the CNS area and age^6^. Accumulation of HA in the epithelial, submucosal and smooth muscle intestinal layers and in blood vessels within the submucosal layer has been observed both in experimental rodent models of colitis and in the intestine of patients with IBD^7,8^, as well as in other inflammatory pathological conditions^9,10^. Due to its exceptional length and high degree of hydration, HA plays an essential role in tissue hydration, lubrication and stability, and data claim also for its anti-inflammatory action^9,11^. Long HA polymers, with high molecular weight, may impede development of inflammatory responses by recruiting different receptors, such as CD44 and toll-like receptors (TLR) 4 and 2, towards the cell membrane^9^. Interestingly, in a mouse model of dextran sodium sulfate-induced colitis HA participated to epithelium repair through TLR4 receptors^12^. HA signaling is, however, strictly dependent upon its molecular weight, since during pathological states, including chronic inflammatory diseases, long HA polymers are cut by hyaluronidases into small fragments, which may promote immune cell activation as well as production of pro-inflammatory cytokines, thus favoring an increased inflammatory response^9^. Regarding IBD, the majority of studies have focused on the involvement of HA in the development of fibrotic tissue within the submucosal and *muscularis propria* layers and on its chemoattractant action for leukocytes in both layers^5,8^. However, no information is available on the possible involvement of HA in myenteric neuron derangement in this condition. In this study, we histochemically investigated the presence of HA in the rat colon myenteric plexus of normal animals and after 2,4-dinitrobenzene sulfonic acid (DNBS)-induced colitis by means of a biotin-labeled HA-binding protein (HABP). HA levels in the colonic wall have been measured by high performance liquid chromatography. The expression of hyaluronan synthase 2 (HAS2), the more abundant of HA synthase isoforms, has also been analyzed.

## Results

### Assessment of colitis

Body weight was significantly reduced in rats after intracolonic administration of DNBS compared to non-inflamed controls (Supplementary Fig. 1, panel A). Six days after DNBS administration, the distal colon was thickened and ulcerated with evident regions of transmural inflammation, adhesions between the colon and other intra-abdominal organs were often present (Supplementary Fig. 1, panel B) and the bowel was occasionally dilated. A twenty-fold increase in macroscopic damage score was observed in DNBS-treated animals in comparison with controls (Supplementary Fig. 1, panel C). Faecal consistency did not significantly change in the DNBS-treated group versus control group (Supplementary Fig. 1, panel D).

Distal colonic cross-sections of control animals showed normal morphology features: a compact epithelium, well-formed crypts and an underlying thin layer of smooth muscle cells forming the *muscularis mucosae*, a submucosal layer containing vascularized loose connective tissue and ganglia of the submucosal plexus and a thick external smooth muscle layer of the *muscularis propria* (Supplementary Fig. 2, panel A). Myenteric ganglia, between the circular and longitudinal muscle of the *muscularis propria*, were compact and formed by healthy neuron and glial cells (Supplementary Fig. 3, panel A).

In distal colon samples obtained from DNBS-treated rats, both mucosa and serosal epithelium displayed prominent morphological abnormalities (Supplementary Fig. 2, panel B). The mucosal surface was irregular and crypt architecture was profoundly altered. The submucosa and *muscularis propria* had significantly increased thickness and showed prominent leukocytes infiltration (Supplementary Fig. 2, panel B). Important degenerative changes were also observed in myenteric ganglia, with neurons displaying dots-like structures in the nucleus, cytoplasm vacuolization and irregular nuclear and cellular membrane. Large spaces between muscle cells were also evident (Supplementary Fig. 3, panel B).

After DNBS treatment, myeloperoxidase (MPO) activity significantly increased (P < 0.01) in mucosa-deprived rat colonic segments versus compared to control animals, suggesting the occurrence of inflammation-induced massive neutrophil infiltration into the intestinal wall (Supplementary Fig. 2, panel C).

### Localization of HA in the rat colon myenteric plexus

**Figure 1:**
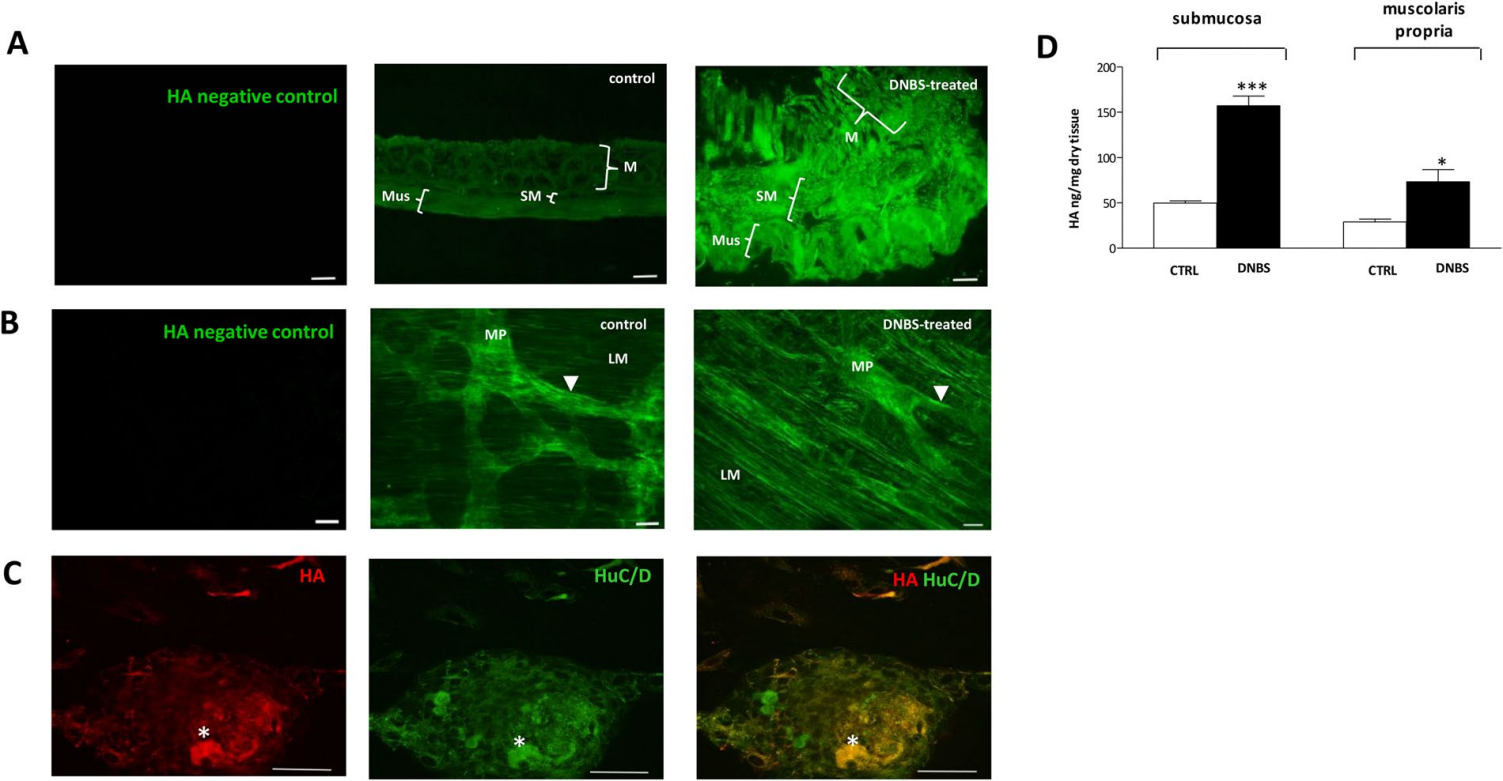
HA staining and levels in the rat colon after DNBS treatment. The immuno-localization of HA was performed on paraffin-embedded tissue sections, colonic whole-mount preparations and primary cultures of myenteric ganglia, using a biotin-labeled HA-binding protein (HABP), which is able to localize HA in tissues by streptavidin conjugation with an appropriate fluorophore. (**A**) HABP staining in rat colon cross-sections obtained from control (vehicle-treated animals) and DNBS-treated animals and relative negative control, bar 100 µm. M, mucosa; SM, submucosa; Mus, *muscolaris propria* (**B**) HABP staining in rat colon LMMP whole-mount preparations from control and DNBS-treated animals and relative negative control, bar 50 µm. LM, longitudinal muscle; MP, myenteric plexus. Arrowhead indicates interconnecting fiber strands in the myenteric plexus. (**C**) Rat small intestine myenteric ganglion in culture double-stained with HABP and and for the pan-neuronal marker HuC/D. Asterisk indicates neurons displaying HA staining. Bar 50 μm. (**D**) HA levels measured in colonic submucosal and *muscularis propria* layers from control (empty bars) and DNBS-treated animals (solid bars). Quantitative analysis of HA was performed by HPLC with fluorimetric detection of derivatized disaccharides obtained after isolation and successive degradation of HA from the submucosal and *muscularis propria* layers. Values are expressed as mean ± S.E.M. of 4 experiments. *P < 0.05 and ***P < 0.001 vs control animals by Student’s t test.

**Figure 2:**
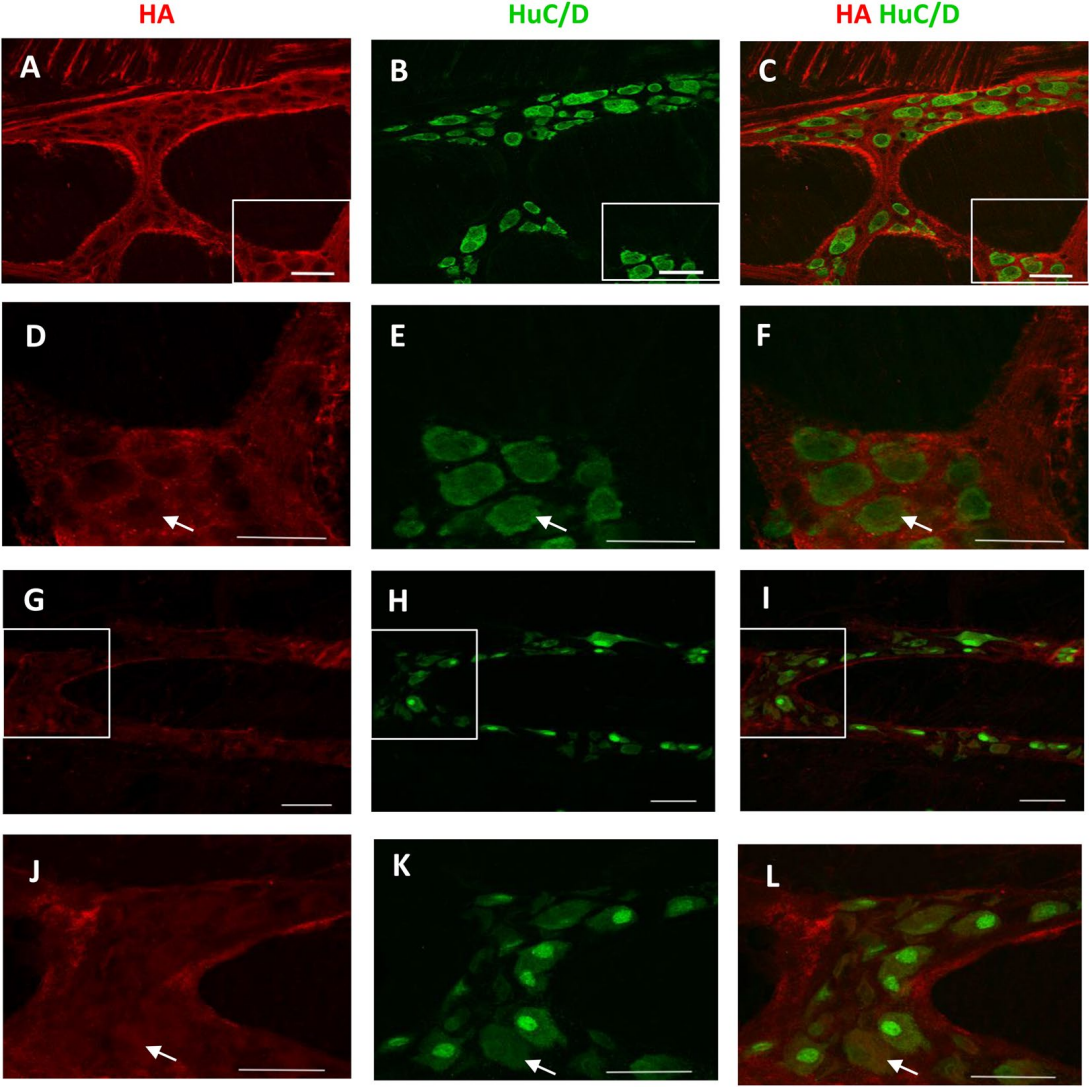
Co-localization of HA with HUC/D in the colonic myenteric plexus of control and DNBS-treated rats. Confocal images showing co-localization of HABP with HuC/D in control (**A–C**) and DNBS-treated (**G–I**) colonic LMMP whole-mounts. In control preparations, HABP faintly stained the soma of myenteric neurons (arrow) and more intensely the perineuronal space contributing (insets, panels **D–F**). After DNBS-treatment, HABP immunofluorescence was still found in the soma of myenteric neurons (arrow) and the well organized perineuronal staining was significantly altered (insets, **J–L**). Bar 50 μm.

**Figure 3:**
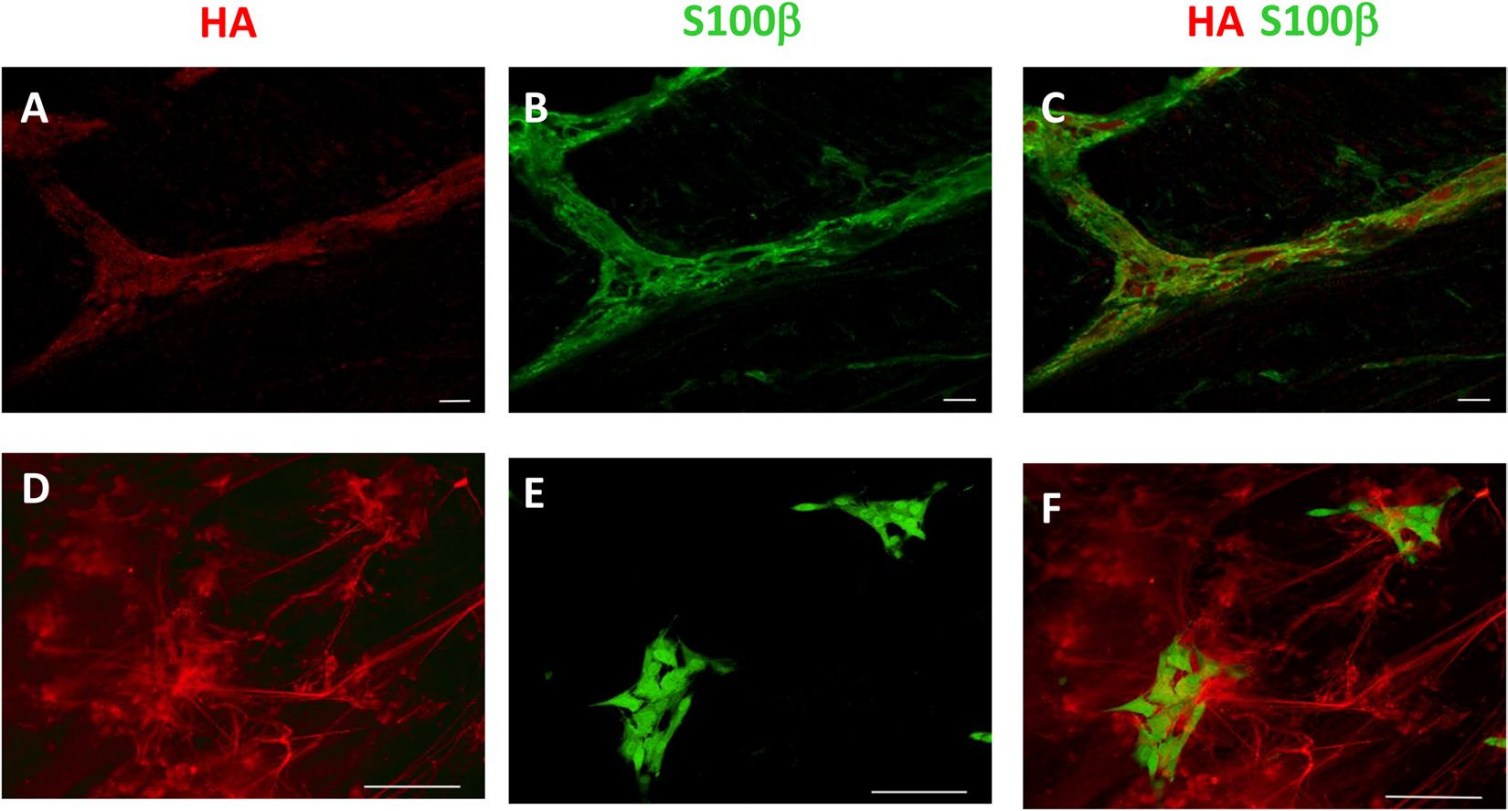
Absence of co-localization between HABP and the glial marker S100β in the rat colon. (**A–C**) Confocal images of a colonic LMMP whole-mount preparation double-stained with HABP and S100β. (**D–F**) Confocal images of rat small intestine myenteric ganglia primary cultures double-stained with HABP and S100β. Bar 50 μm.

In control colonic cross-sections, the distribution of HA was regular in the epithelial crypts, the underlying submucosal and *muscularis propria* layer with the myenteric plexus, as evidenced by HA binding protein (HABP) staining. (Fig. 1, panel A). In longitudinal muscle myenteric plexus (LMMP) whole mount preparations, HA staining was particularly intense on the surface of myenteric ganglia (Fig. 1, panel B). In addition, a faint HA labeling was found in myenteric neuron cytoplasm, as demonstrated by co-staining with the neuronal marker HuC/D. A more intense and well organized HA labeling was found in the perineuronal space, surrounding myenteric neurons (Fig. 2, panels A–F). Intense HA staining was also found along interconnecting fiber strands (Fig. 1, panel B). The ability of HABP to label myenteric neurons suggests that, in the myenteric plexus, neuronal cells may represent a source for HA. To confirm this hypothesis we evaluated HABP binding in primary cultures of rat small intestine myenteric ganglia. In these preparations, HA staining was particularly intense in the soma of some neurons, as confirmed by co-staining with HuC/D (Fig. 1, panel C). In addition, cable-like HA structures departing from neurons were observed to connect different cellular aggregates (Fig. 3, panels D and F). In myenteric ganglia, enteric glial cells do not contain HA, as suggested by the absence of co-staining between HA and the enteric glial cell marker, S100β, both in colonic LMMPs (Fig. 3, panels A–C) and in primary cultures of myenteric ganglia (Fig. 3, panels D–F).

### HA levels are upregulated after DNBS-induced colitis

In agreement with data obtained with cross-sections of DNBS-treated rat colon^13^, in colonic whole-mounts, DNBS-induced inflammation was associated with a reduction in the total number of colonic myenteric neurons, which displayed a reduced soma area compared to control preparations (Supplementary Figure 3, panels C–F). In addition, after DNBS treatment, HuC/D immunoreactivity was faint in the cytoplasm of the majority of myenteric neurons, and a number of neurons showed increased HuC/D staining in the nucleus relative to the cytoplasm (Supplementary Figure 3, panels C–D).

After DNBS treatment, HA staining increased in both colonic cross-sections and LMMPs compared to control animals (Fig. 1, panels A-B). Abundant HABP staining was observed in the smooth muscle cell layer of the *muscularis propria* (Fig. 1, panels A–B) and in the submucosal layer. Accordingly, HA levels significantly increased (p < 0.001 and p < 0.05, respectively) in both layers versus values obtained in control preparations (Fig. 1, panel D). In preparations obtained from DNBS-treated animals, the structured HA distribution within myenteric ganglia disappeared. In particular, perineuronal HA was less evident than in control preparations. HA labeling was still present in the cytoplasm of myenteric neurons, on ganglia surface and along interconnecting fibers (Fig. 1, panels A and B and Fig. 2, panels G–L).

### HAS2 is expressed in myenteric neurons of the rat colon

**Figure 4:**
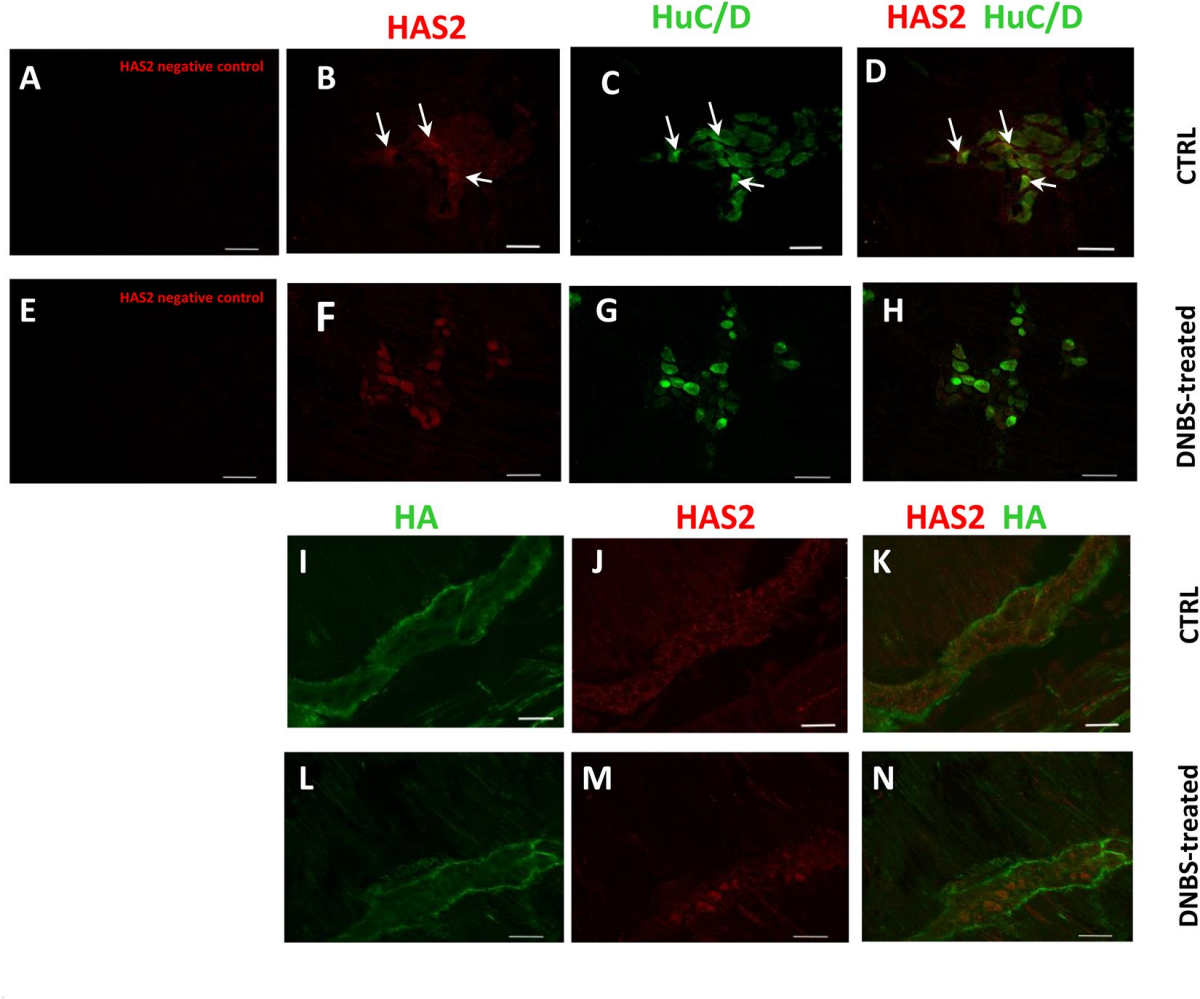
HAS2 and HuC/D co-localization in the rat colon myenteric plexus of control and DNBS-treated animals. (**A–H**) Confocal images showing co-localization of HAS2 with HuC/D in LMMP whole-mount preparations obtained from in control (**B–D**; arrows indicate myenteric neurons co-staining for HAS2 and HuC/D) and after DNBS-treatment (**F–H**). (**I–N**) HAS2 and HABP co-localization in the rat colon myenteric plexus of control and DNBS-treated animals. Co-localization of HAS2 with HABP in LMMP whole-mount preparations of control (**I–K**) and DNBS-treated animals (**L–N**). Panels A and E show negative controls for HAS2 staining in control and DNBS-treated preparations, respectively. Bar 50 μm.

**Figure 5:**
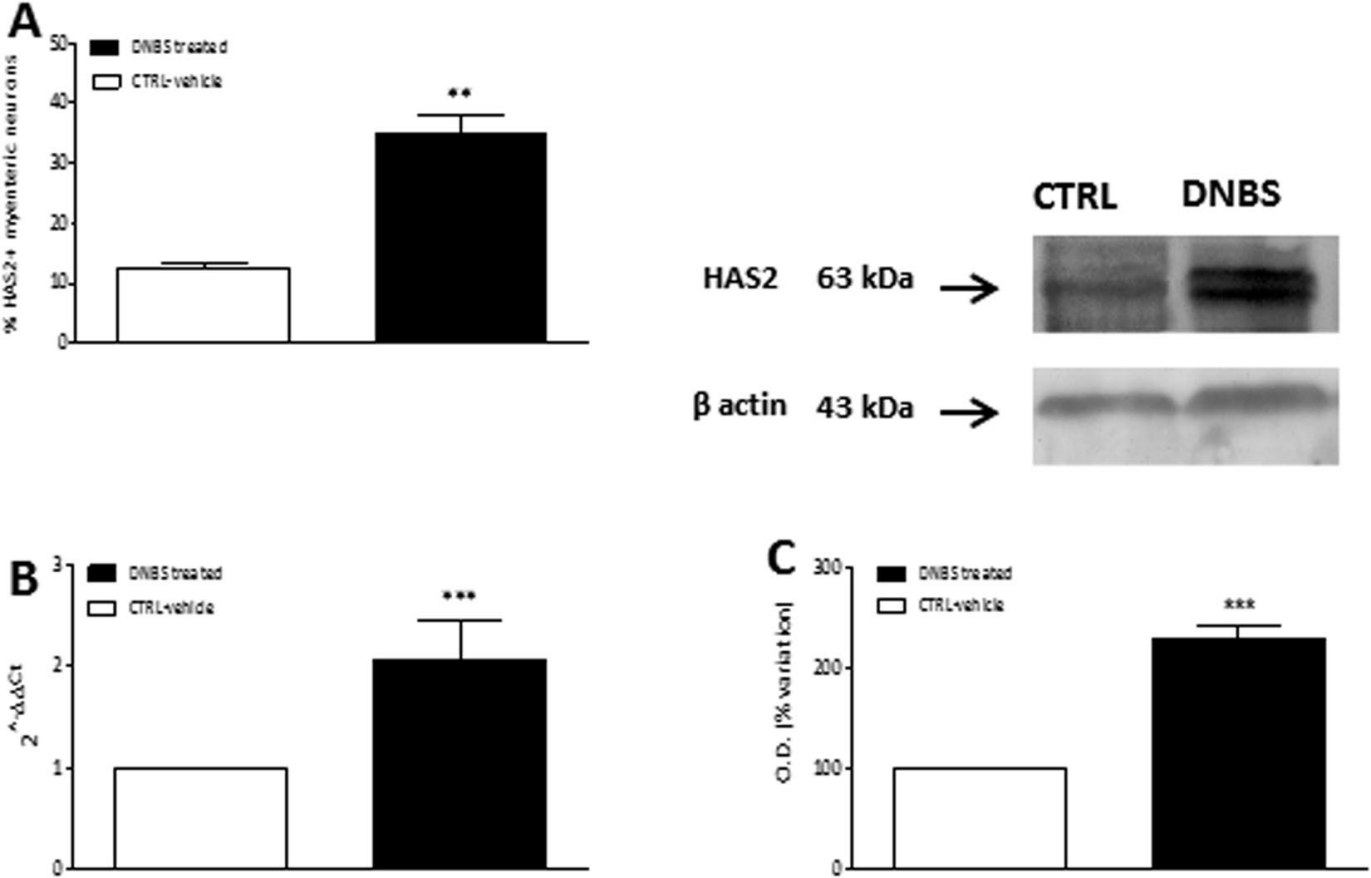
HAS2 expression in myenteric ganglia. (**A**) Percentage of myenteric neurons per mm^2^ co-staining for HuC/D and HAS2, in rat colon obtained from control (vehicle-treated, empty bars) and from DNBS-treated animals (solid bars). Values are expressed as mean ± S.E.M. of 3 experiments. (**B**) RT-PCR quantification of HAS2 transcripts in control (empty bars) and in DNBS-treated animals (solid bars). Values are mean ± S.E.M. of 8 experiments of the percentage variation of relative gene expression with respect to values obtained in control animals. The relative gene expression was determined by comparing 2^−ΔΔCt^ values normalized to β-actin. (**C**) HAS2 protein expression analyzed in lysates fractions of LMMPs obtained from control (empty bars) and DNBS-treated animals (solid bars). Blots representative of immunoreactive bands for HAS2 and β-actin in the different experimental conditions are reported on top of each panel. Samples (100 μg) were electrophoresed in SDS-10% polyacrylamide gels. Numbers at the margins of the blots indicate relative molecular weights of the respective protein in kDa. Values are expressed as mean ± S.E.M. of 4 experiments of the percentage variation of the normalized optical density (O.D.) obtained from DNBS-treated preparations with respect to values obtained in control samples. **P < 0.01 and ***P < 0.001 vs control animals by Student’s t test.

Immunofluorescence experiments revealed the presence of hyaluronan synthase 2 (HAS2) in rat distal colon myenteric plexus. Double labeling with either HuC/D or HABP, showed HAS2 immunoreactivity in the cytoplasm of a small percentage of myenteric neurons (12.63 ± 0.58%, n = 3) (Fig. 4, panels B-D, I-K; Fig. 5, panel A). In addition, HAS2 antibody stained neuronal profile discontinuously within myenteric ganglia (Fig. 4, panels I-K). In colonic LMMPs of control rats, qRT-PCR and western blotting investigations revealed the presence of both transcript and protein of HAS2 (Fig. 5, panels B–C).

### DNBS-induced colitis upregulates HAS2 in myenteric neurons

In colonic LMMPs of DNBS-treated rats the number of HAS2 immunopositive neurons (Fig. 4, panels F–H and L–N) significantly increased (p < 0.01) versus values obtained in controls (Fig. 5, panel A). In myenteric ganglia, HAS2 staining around neuronal profile was less evident in preparations from treated rats than in controls (Fig. 4, panels L–N). After DNBS-induced colitis, the levels of HAS2 transcript and protein were significantly higher (p < 0.001) compared to control animals (Fig. 5, panels B–C). In LMMP preparations obtained from control rats, HAS2 specific antibody revealed one band at 63 kDa, while a double band could be observed at 63 kDa in preparations obtained from DNBS-treated mice, which may result from post-translational modifications of the protein induced by inflammation^9^.

## Discussion

In this study, morphological investigations, resorting to a fluorescently labeled hyaluronan binding protein (HABP) as a marker to detect HA, and biomolecular data provide evidences that HA is produced by myenteric neurons and is abundantly present in myenteric ganglia. Our findings are the first demonstration that a glycosaminoglycan (GAG) component of the extracellular matrix (ECM) may participate in the formation of a pericellular coat of condensed matrix surrounding myenteric neurons, similar to the perineuronal net associated with some classes of neurons within the central nervous system (CNS)^14^.

After DNBS-induced colitis, the HA containing perineuronal structure is significantly altered, resulting in the loss of the structured organization observed in control preparations. In addition, HA levels significantly increase in the *muscularis propria* containing the myenteric plexus suggesting that the GAG may participate to colonic neuromuscular derangement during inflammation.

Immunohistochemical reports have previously demonstrated that ECM molecules, such as laminin, type IV collagen, nidogen, heparin sulfate proteoglycans and fibronectin, are present in or nearby the basement membrane that surrounds myenteric ganglia^15^. In accordance we found strong HA staining on myenteric ganglia surface, indicating that HA may contribute to the external architecture of enteric ganglia. However, in contrast with previous reports, that could not detected ECM molecules within myenteric ganglia^15^, in this study we observe a strong HA staining in the perineuronal space surrounding myenteric neurons. In recent years, several studies have documented that a large part of the neuronal tissue in the CNS is occupied by a soft ECM, mainly composed of GAG, in the form of HA or as part of specific proteoglycans^16^. In particular, a special and stable extracellular matrix structure, called the perineuronal net, surrounds certain types of neurons^16^. Chondroitin sulfate proteoglycans, HA and its link proteins HAPLN1 and HAPLN4 as well as the large glycoprotein tenascin-R, are the main components of the perineuronal net, whose main functions are the regulation of ion homeostasis around active neurons, stabilization of synapse and participation to neuronal plasticity^6,17^. Although astrocytes represent a well established cellular source for HA in the brain^18^, studies carried out in dissociated cortical neurons devoid of astrocytes have suggested the potential role for neuronal HA production^19^. Our finding that a perineuronal sheath, composed of HA, envelops myenteric neurons represents a further demonstration of the similitude between the CNS and enteric nervous system (ENS), whose structure and organization resemble those of the CNS more than any other part of the peripheral nervous system^3^.

In both colonic whole mounts and in primary cultures of myenteric ganglia, myenteric neurons co-express HA and the neuronal marker HuC/D, suggesting that this cell type may represent a source for the GAG. However, in colonic whole mounts HABP only faintly stains the cytosol of myenteric neurons, while a strong labeling can be found in the soma of some myenteric neurons in culture. This discrepancy may depend upon the presence of trophic factors in the culture medium used to grow isolated myenteric ganglia, which may favor a higher HA turnover^20,21^.

Another important data obtained in this study is that myenteric neurons express HAS2, the main isoform of the three transmembrane HA synthases (HAS1–3) in adult cells^9^. Interestingly, the presence of both HAS2 mRNA and protein has recently been demonstrated in developing rat cortical neurons, consolidating the hypothesis that neurons have the capacity to synthesize HA^22^. HA may sustain the formation of the perineuronal sheath enveloping myenteric neurons by binding to its receptors, such as CD44 or RHAMM located on neuronal surface, or directly via HASs, which can retain HA on cell surface^23^. Both hypotheses need to be confirmed with further investigations. The absence of co-staining between HABP and the glial cell marker, S100 β, in both colonic whole-mounts and isolated myenteric ganglia would exclude the presence of HA in enteric glia.

DNBS treatment induced a drastic HA redistribution in the colonic neuromuscular compartment. As observed in human IBD colon tissue and in murine dextran sodium sulfate (DSS)-induced colitis, HA accumulates in the *submucosal* and *muscularis propria* layers^8,24^. From a structural viewpoint, our data, show a rearrangement of HA distribution pattern with a transition from a well-defined HA matrix to dense deposits in all of the intestinal layers, as observed in the DSS-treated mouse colon^8^. A peculiar feature of the inflamed colon is submucosal and smooth muscle thickening with increased deposition of connective fibers associated with enhanced production of collagen and non-collagen extracellular matrix components^13,25^. We cannot exclude that increased HA levels in both layers during inflammation may take part in the fibrotic process, as suggested in other peripheral organs and in vascular smooth muscle cells^5,10,26,27^.

In contrast with previous studies, we here report that inflammation may induce considerable changes in HA distribution also in myenteric ganglia. At this level, the perineuronal HA envelope is significantly reduced, while HABP staining in neuronal cytosol and ganglia surface is still present. Evidences obtained in both humans and experimental animal models, suggest that the ECM perineuronal net retains a neuroprotective role under conditions of neurodegenerative diseases, or against excitotoxicity, amyloid-β toxicity deposition or oxidative stress, in selective vulnerable neuron types^16^. Hence, we cannot exclude that alterations in perineuronal HA deposition may contribute to changes in myenteric neuron structure and function during an inflammatory challenge. Consistently with previous reports, after DNBS treatment, myenteric ganglia display significant changes, including a drastic reduction of HuC/D positive neurons. Several signs of neuronal degeneration are also observed, including the presence of nuclear aggregates, cytosolic vacuolization, smaller area and changes in HuC/D immunoreactivity distribution^13^. The increase in HuC/D nuclear staining with respect to the cytoplasm is indicative of myenteric neuron derangement, which may occur in different pathological conditions, including intestinal ischemia/reperfusion injury^28,29^. We cannot exclude that the degradation of a perineuronal HA sheath might contribute to alter myenteric neuron excitability, as observed after degradation of the perineuronal net in cortical mouse slices^30^. In a recent study carried out on a HAS3 deficient mouse model, reduction of HA deposition in the CA1 stratum pyramidale of the hippocampus was associated with a reduced extracellular space volume and increased epileptogenic activity^31^.

Alteration of HA distribution after experimentally-induced colitis was associated with up-regulation of HAS2 expression in myenteric neurons. Interestingly, expression of HA synthetic enzymes, such as HAS2 and HAS3, but not of HAS1, enhanced in mouse colon epithelium after destran-sodium sulphate-induced colitis^12^. Accordingly, in human intestinal microvessel endothelial cells and submucosal smooth muscle cells, HAS2 and HAS3 are upregulated in response to stressor or proinflammatory stimuli^8,24^. In these latter studies, increased HAS expression was correlated with the ability of HA to behave as an adhesion molecule secreted by microvessel endothelial cells and submucosal smooth muscle cells in order to recruit mononuclear leukocytes via their CD44 receptors^5^. We cannot exclude that HAS2 increased expression in myenteric neurons during experimentally-induced colitis is finalized to retain inflammatory cells in the proximity of myenteric ganglia, since we observed prominent leukocyte infiltration. The interplay between myenteric neurons and immunocytes may be fundamental in the remodeling of the neuronal network in response to a neuromuscular damage during gut inflammation. Both myenteric neurons and immunocytes may regulate one another’s functions by releasing a complex set of cytokines, neurotransmitters and hormones. Neuronal activation can lead to degranulation of mast cells and recruitment of neutrophils to the area^32^. HA produced by HAS2 in myenteric neurons, may therefore play a role in recruiting immunocytes nearby myenteric ganglia during intestinal inflammation^5^. This hypothesis is substantiated by the ability of myenteric ganglia in culture to produce HA “cable like” structures, which confer to HA the ability to attach to serum components, such as the heavy chain of inter-alpha trypsin inhibitor, which are known to increase the adhesiveness of HA to leukocytes^33^. Further studies aiming to evaluate the possible role of HASs in the myenteric plexus, as well as the use of conditional knock-out animal models^34–36^ of the different HASs in myenteric neurons, may give a more complete view on HA involvement in neuromuscular dysfunctions associated with development of colitis.

In conclusion, in this study we provide evidence that myenteric neurons may produce HA which retains a homeostatic role by contributing to the formation of an extracellular matrix basal membrane enveloping the surface of myenteric ganglia as well as a perineuronal net surrounding myenteric neurons. After DNBS-induced colitis this well organized HA structure is highly altered, especially within myenteric ganglia. This alteration is associated with increased neuronal HAS2 expression and may participate to myenteric neuron derangement underlying changes in motor function. We cannot exclude that modulation of HA production during intestinal inflammation may ameliorate intestinal motility patterns which represent a remarkable cause of IBD symptoms.

## Materials and Methods

### Animals

Male Sprague-Dawley rats (weight 250–300 g, Envigo, San Pietro al Natisone, Udine, Italy), were housed under controlled environmental conditions (temperature 22 ± 2 °C; relative humidity 60–70%) with free access to a standard laboratory chow and tap water, and were maintained at a regular 12/12-h light/dark cycle. Their care and handling were in accordance with the provision of the European Union Council Directive 2010/63, recognized and adopted by the Italian Government (Decree No. 26/2014). The protocol was approved by the Animal Care and Use Ethics Committee of the University of Insubria and of the University of Pavia.

### DNBS-induced colitis

Experimental colitis has been induced in accordance with the method described by Vasina *et al*.^37^, by administration of a single dose (30 mg) of 2,4-dinitro-benzene-sulfonic acid (DNBS, ICN Biomedicals, CA, USA) dissolved in 0.25 ml of 50% ethanol and administered, under isofluorane anaesthesia, via a polyethylene PE-60 catheter into the colon 8 cm proximal to the anus. This dose was selected on the basis of a previous study showing that it evoked adequate inflammation without causing unnecessary distress and suffering to the animals, with a mortality rate of 0%. Controls were administered 0.25 ml of 50% ethanol (vehicle). Animals were euthanized 6 days after the induction of colitis, when the intestinal inflammatory process is maximal^37^, and the distal colon was removed, opened longitudinally over the mesenteric line and washed with a physiological Tyrode’s solution (in mM: 137 NaCl; 2.68 KCl; 1.8 CaCl2.2H2O; 2 MgCl2; 0.47 NaH2PO4; 11.9 NaHCO3; 5.6 glucose). DNBS-treated and control rats were kept in separated cages during the study. DNBS-induced experimental colitis in rats was chosen since, the inflammatory response develops rapidly (6 days) and shares many features with the response observed in human IBDs^13^. Possible physiological and behavioral changes were monitored throughout the treatment period (i.e., changes in body weight, respiration, occurrence of diarrhea, alterations of posture and in the appearance of the coat) to evaluate suffering and distress.

### Assessment of colonic damage

Colonic damage was evaluated macroscopically and histologically. Macroscopic colonic damage was evaluated according to standard procedures^38^. Briefly, the criteria adopted were the following: presence of adhesions between the colon and other intra-abdominal organs (0 = none, 1 = mild, 2 = major); consistency of colonic faecal material (as an indirect marker of diarrhea) (0 = formed, 1 = loose, 2 = liquid); thickening of the colonic wall, presence and extension of hyperemia and macroscopic mucosal damage [0 = no damage; 1 = hyperemia; 2 = presence of an ulcer; 3 = ulcer + inflammation; 4 = two or more ulcers; 5 = major damage (presence of necrosis <2 cm); 6 = very severe damage (presence of necrosis >2 cm)]. Ethanol, used as a vehicle to break the mucosal barrier, thus allowing DNBS penetration into the bowel wall, per se, had no effect on the parameters measured 6 days after induction of colitis. Microscopic evaluation of the damage was histologically investigated. To this end, full-thickness samples of distal colon obtained from both DNBS-treated and control rats were fixed with 4% formaldehyde in acetate buffer 0.05 M for 24–48 h and successively embedded in paraffin. Hematoxylin and eosin (HE) histological staining was carried out on seven-micron-thick sections and observed under a light microscope (Nikon Eclipse Ni; Nikon, Tokyo, Japan). Data were recorded using a DS-5M-L1 digital camera system (Nikon Corporation, Tokyo, Japan).

### Myeloperoxidase activity

Myeloperoxidase (MPO) was measured according to Filpa *et al*.^39^, in order to assess the development of an inflammatory state caused by neutrophil infiltration. Briefly, mucosa-deprived intestinal samples were suspended in ice cold potassium phosphate buffer (50 mM, pH 6.0) containing 0.5% hexadecyl trimethylammonium bromide (HTAB) and homogenized (50 mg/ml). After centrifugation (14 000 rpm, 20 minutes, 4 °C), an aliquot of the supernatant fraction (34 μl) was mixed with 986 μl of the HTAB-phosphate buffer containing 0.167 mg/ml O-dianisidine dihydrochloride with hydrogen peroxide (0.0005%). Changes in the rate of absorbance were spectrophotometrically recorded at 460 nm. MPO activity was expressed in units (U),defined as the amount of enzyme that degrades 1μmol/min of hydrogen peroxide at 25 °C. Experiments were performed four times, and results were expressed in U/mg wet tissue weight.

### Immunofluorescence

The immuno-localization of HA was performed on paraffin-embedded tissue sections, colonic whole-mount preparations and primary cultures of myenteric ganglia, using a biotin-labeled HA-binding protein (HABP, Seikagaku Co, Japan). HABP recognizes HA saccharidic sequences and is able to localize HA in tissues by streptavidin conjugation with an appropriate fluorophore^40^. Hyaluronan synthase 2 (HAS2) distribution in myenteric ganglia was also evaluated by immunofluorescence on colonic whole-mount preparations.

#### Paraffin sections

Paraffin cross sections (7 μm) of both control and DNBS-treated rat colon were treated for 30 minutes with PBS containing 2% bovine serum albumin (BSA) before biotin-labeled HABP overnight incubation (4 °C). After washing, incubation with a suitable streptavidin FITC-conjugated antibody was performed for 60 minutes in a dark humid chamber. Concentrations of HABP and streptavidin FITC are reported in Table 1. PBS buffer used for washing steps and HABP dilutions contained 2% BSA. Control samples were incubated with BSA-containing PBS. Coverslips were mounted with Citifluor mounting medium and then observed under a fluorescent microscope (Nikon Instruments).

**Table 1:** Primary and secondary antisera and their respective dilutions used for Western Blot (WB) assay and immunohistochemistry (HC).

Antiserum	Dilution (WB)	Dilution (HC)	Source	Host species
Primary antisera				
HUC/D	______	1:100	Molecular Probes (A-21272)	Mouse
S-100	______	1: 200	Dako (Z0311)	Rabbit
HAS2	1:200	1:100	Santa Cruz (sc34068)	Goat
β-actin	1:2000	_______	Santa Cruz (sc1616)	Goat
Secondary antisera & streptavidin complexes				
Anti-rabbit Alexa Fluor 488	______	1:200	Molecular Probes (A21206)	Donkey
Anti-goat Cy3	______	1:400	Jackson (705-165-147)	Donkey
Cy3-conjugated streptavidin	______	1:500	Amersham (PA43001)	
FITC-conjugated straptavidin	______	1:200	Molecular Probes (SA1001)	
F(ab’)_2_ Anti-mouse IgG (H + L) biotin	______	1:300	Caltag laboratories (M35015)	Goat
F(ab’)_2_ Anti-rabbit IgG (H + L) biotin	______	1:300	Caltag laboratories (L43015)	Goat
Anti-goat IgG HRP peroxidase conjugated	1:5000 1:20000	______	Santa Cruz (sc2020)	Donkey

**Supplying companies:** Amersham, GE Healthcare, Buckinghamshire, UK; Caltag Laboratories, Invitrogen, Burlingame, CA, USA; Dako, DK-2600 Glostrup, Demark; Jackson Immuno Research Laboratories, Inc., Baltimore, USA; Molecular Probes, Invitrogen, Carlsbad, CA, USA; Santa Cruz Biotechnology, Inc., CA,USA.

#### Rat colonic whole-mounts

For whole-mounts immunolabelling, distal rat colon segments were filled with 0.2 mol/l sodium phosphate-buffer (PBS): (in M: 0.14 NaCl, 0.003 KCl, 0.015 Na_2_HPO_4_, 0.0015 KH_2_PO_4_, pH 7.4) containing 4% formaldehyde and 0.2% picric acid for 4 hours at room temperature (RT). The longitudinal muscle with the attached myenteric plexus (LMMP) was gently removed from the rest of the intestinal wall and processed for whole-mount staining as described by Giaroni *et al*.^41^. After blocking non-specific sites using PBS with added 1% Triton X-100 and 10% normal horse serum (NHS) (Euroclone, Pero, Italy) for 1 hour, LMMPs were incubated with either primary antibody (HuC/D neuronal marker, or S100β, glial marker, or HAS2, Table 1) or with biotin-labeled HABP (1:40), overnight at RT. Then addition of an appropriate secondary antibody was performed for 2 hours at RT. Double-labeling was performed by incubating either a second primary antibody or HABP with the same procedure. LMMPs were mounted on glass slides using Vectashield mounting medium with DAPI (Vector Lab, Burlingame, CA, USA). Total neuron number per ganglion area was expressed as the ratio between the number of HuC/D positive neurons within the ganglion and the total ganglion area (µm^2^) measured with Image J NIH image software (http://imagej.nih.gov/ij)^42^. Neuronal cell body area was measured with Image J. Confocal images of 15 ganglia captured from preparations obtained from vehicle-treated and DNBS-treated animals, respectively, were used both for neuronal cell counting and area measurement (the number of neurons counted per animal was 360, 337 and 397 for controls, and 338, 412 and 583 for DNBS-treated preparations). To establish the proportion of HAS2 expressing myenteric neurons, the number of HAS2 immunoreactive neurons that co-localized with HuC/D were counted and expressed as percentage of the total number of HuC/D positive neurons^41^. A total of 10–15 ganglia were sampled from LMMP preparations obtained from 3 animals for each experimental group. Negative controls and interference control staining were evaluated by omitting of both primary and secondary antibody, and by incubating colonic whole-mounts with non-immune serum from the same species in which the primary antibodies were raised. In all of these conditions, no specific signal was detected. Preparations were analyzed using a Leica TCS SP5 confocal laser scanning system (Leica Microsystems GmbH, Wetzlar, Germany) and pictures were processed with Adobe-Photoshop CS6S software.

#### Primary cultures of myenteric ganglia

Primary cultures of myenteric ganglia were prepared from adult rat small intestine. The small intestine was chosen to fulfil the reduction principle of good laboratory animal procedures, since a higher yield of myenteric ganglia can be obtained from a single animal small intestine with respect to the distal colon. Segments (20 cm long) of the small intestine, 3 cm oral to the ileo-caecal junction, were isolated, and rinsed with a physiological ice-cold Tyrode’s solution. Myenteric ganglia cultures were obtained by mechanical and enzymatic digestion of fragmented intestinal segments constituted of LMMPs according to Carpanese *et al*.^43^. After dissociation, 3 × 10^4^ cells per well were seeded on poly-L-lysin (100 ng/L) pre-coated glass cover slips (12 mm in diameter) in 24-well dishes and grown in an incubator (37.5 °C, 5% CO_2_). Myenteric ganglia cultures were cultured for six days in primary cultures and the medium was changed every two days. For immunofluorescence staining, cells on coverslips were fixed in PBS containing 4% formaldehyde for 10 min at 37 °C. After blocking non-specific sites with PBS containing 5% normal horse serum (Euroclone) and 0.1% Triton X-100 for 1 h at room temperature, preparations were double-labelled with HABP and with either an anti-HuC/D or an anti-S100β antibody. Preparations were mounted on glass slides and analyzed by confocal microscopy. Negative controls were evaluated as described for whole-mounts immunolabelling.

### Real time quantitative RT-PCR

To evaluate the influence of DNBS-induced colitis on HAS2 mRNA levels, extraction of total RNA from rat colon LMMPs was extracted with TRIzol (Invitrogen) and treated with DNase I (DNase Free, Ambion) to remove possible traces of contaminating DNA. 2.5 μg of total RNA were then retrotranscribed using the High Capacity cDNA synthesis kit (Applied Biosystems, Life Technologies, Grand Island, NY, USA). Quantitative RT-PCR was performed with an Abi Prism 7000 real-time thermocyclator (Applied Biosystems) using the TaqMan® Gene Expression Mastermix (Applied Biosystem) following manufacturer’s instructions. TaqMan gene expression assays were used to detect HAS2 (Hs00193435_m1) and the housekeeping gene β-actin (Hs99999903_m1) levels. The 2^−ΔΔCt^ method^44^ was applied to determine the relative gene expression. Experiments were performed eight times and the effect of DNBS-induced colitis on HAS2 mRNA levels in LMMPs preparations was obtained by comparing the 2^−ΔΔCt^ values with those measured in colonic LMMPs specimens obtained from vehicle-treated control animals.

### Western immunoblot analysis

Colonic LMMPs preparations were used to analyze HAS2 protein level according to the method described by Giaroni *et al*.^45^. Briefly, purified membrane fractions were obtained after successive centrifugations and boiled for 2 min in Laemmli sample buffer and processed as described elsewhere^46^. Membrane incubation with HAS2 primary antibody, was followed by incubation with a horseradish peroxidase-conjugated secondary antisera (Table 1). The antibody/substrate complex was visualized by chemiluminescence using an enhanced chemiluminescence kit (ECL advance Amersham Pharmacia Biotech, Cologno Monzese, Italy). Signal intensity was evaluated by densitometric analysis using Image J NIH image software. β-actin was used as protein loading control. The effect of DNBS-induced colitis on HAS2 protein levels was expressed as the percentage variation of the optical density (expressed in arbitrary units) of HAS2 signal normalized to the respective β-actin in DNBS treated LMMPs compared to controls. Experiments were performed in quadruplicates. The specificity of HAS2 antibody was evaluated by testing its selectivity in NIH3T3 cells (data not shown). Negative controls were performed by omitting the primary antibody.

### Quantification of HA levels in the submucosal and muscularis propria layers

HA levels were measured in the submucosal and *muscularis propria* layers of DNBS-treated and vehicle-treated control animals following the procedures described in Raio *et al*.^40^, with modifications. To obtain the submucosal and *muscularis propria* layers, 3 cm long colonic segments were cut along the longitudinal axis and opened flat on a silgard support. After gently removing the mucosa layer with a small blade, the submucosal layer was separated from the *muscularis propria* under a dissecting microscope. Samples of the submucosal and *muscularis propria* were then lyophilized. HA was purified and digested with hyaluronidase SD in order to obtain Δ disaccharides which were then derivatized with 2-aminoacridone (AMAC) according to Karousou *et al*.^47^. Separation and analysis of AMAC-derivatives of Δ-disaccharides were done with a Jasco-Borwin chromatograph system with a fluorophore detector (Jasco FP-920, λex = 442 nm and λem = 520 nm). Chromatography was carried out using a reversed phase column (C-18, 4.6 × 150 mm, Bischoff) at room temperature, equilibrated with 0.1 M ammonium acetate, pH 7.0, filtered through a 0.45 μm membrane filter. A gradient elution was done using a binary solvent system composed of 0.1 M ammonium acetate, pH 7.0 (eluent A), and acetonitrile (eluent B). The flow rate was 1 ml/min, and the following program was used: pre-run of column with 100% eluent A for 20 min, isocratic elution with 100% eluent A for 5 min, gradient elution to 30% eluent B for 30 min and from 30% to 50% for 5 min. Sample peaks were identified and quantified comparing the fluorescence spectra with standard Δ-disaccharides, using Jasco-Borwin software. Experiments were performed four times and HA levels were expressed as ng of HA per mg of dry tissue.

### Statistical analysis

All data are expressed as mean ± SEM. Statistical significance was calculated with Student’s t test for unpaired data or one way ANOVA with Bonferroni’s post hoc test, using GraphPad Prism (version 5.3 GraphPad software, San Diego, CA, USA). The differences between groups were considered significant at *P* values < 0.05.

## Electronic supplementary material

Supplementary Figures
