# Supplementary material for: Changes in hyaluronan deposition in the rat myenteric plexus after experimentally-induced colitis
Source: Sci Rep. 2017 Dec 15;7:17644. doi: 10.1038/s41598-017-18020-7 (PMC5732300; doi:10.1038/s41598-017-18020-7)
Supplement: Supplementary file 1 — Supplementary Figures [file 41598_2017_18020_MOESM1_ESM.pdf]

Changes in hyaluronan deposition in the rat myenteric plexus after experimentally-induced colitis

Viviana Filpa, Michela Bistoletti, Ilaria Caon, Elisabetta Moro, Annalisa Grimaldi, Paola Moretto, Andreina Baj, Maria Cecilia Giron, Evgenia Karousou, Manuela Viola, Francesca Crema, Gianmario Frigo, Alberto Passi, Cristina Giaroni, Davide Vigetti

Supplementary Figure 1

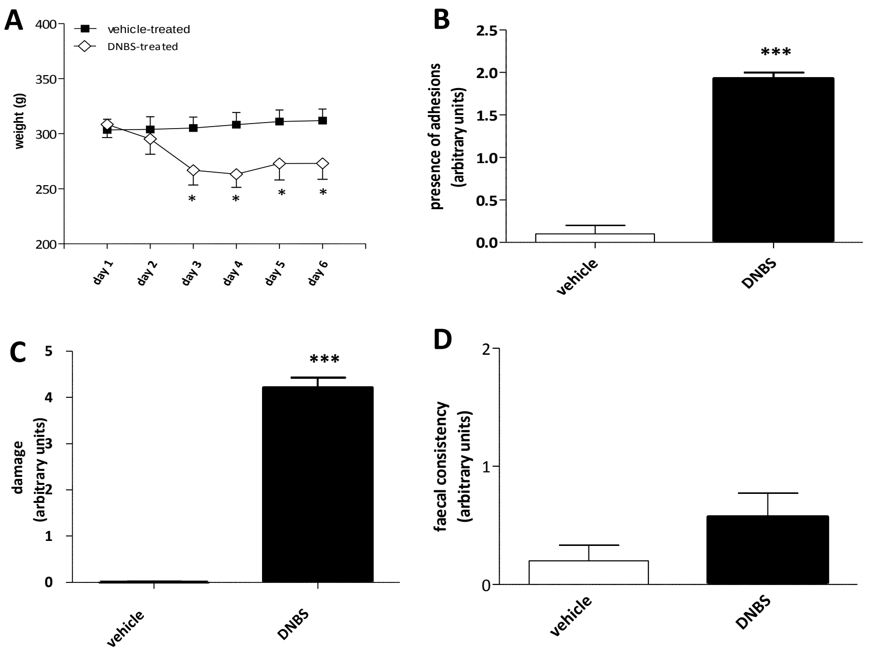

Supplementary figure 1: (A) Body weight change in control (vehicle-treated) and DNBS-treated animals (30 mg/rat). (**B**) presence of adhesions and (**C**) macroscopic damage expressed in arbitrary units in colonic specimens obtained from DNBS-treated animals (solid bar) and from controls (empty bar). (**D**) Faecal consistency in DNBS-treated animals (solid bar) and controls (empty bar). Data are expressed as mean ± S.E.M. n= 7 rats per group. * P<0.01 and by one way ANOVA with Bonferroni's post hoc test and *** P<0.001 by Student's t test vs control .

Supplementary Figure 2

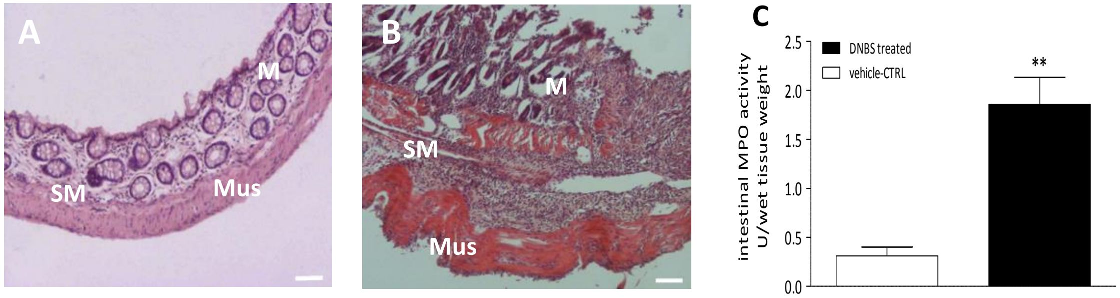

Supplementary figure 2: (**A**) Hematoxylin-eosin (HE) staining highlights the well-preserved cellular and structural morphology in the cross sectioned colon of control (vehicle-treated) rats (bar 100 µm). **(B)** Cross section of colon samples collected from DNBS-treated rat. HE staining highlights the cellular and structural changes in the intestine, including increased thickness of the submucosal and *muscularis propria* layers (bar 100 µm). **M**, mucosa; **SM**, submucosa; **Mus,** *Muscolaris Propria*. (**C**) Myeloperoxidase (MPO) activity measured in mucosa-deprived colonic segments obtained from DNBS-treated (solid bar) and control animals (empty bar). Values are expressed as mean±SEM of MPO activity of 4 experiments. ***P*<0.01 *vs* values obtained in control animals by Student's t test.

Supplementary Figure 3

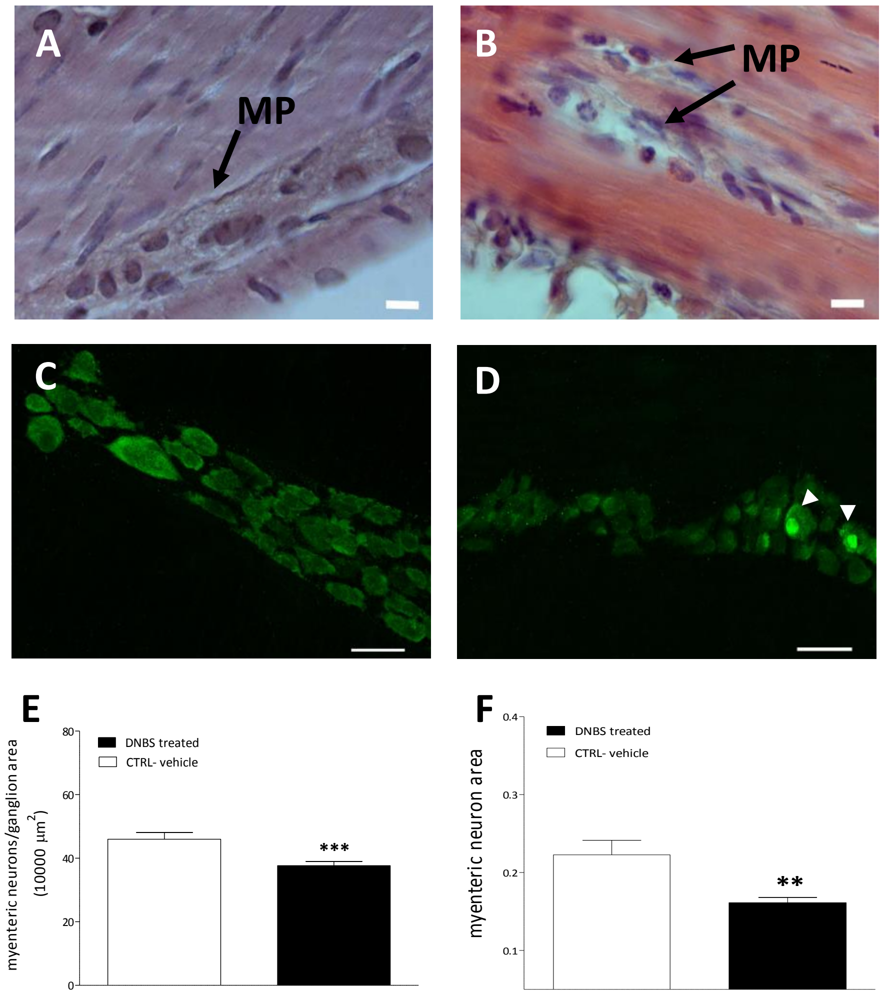

Supplementary figure 3: DNBS-treatment induced changes in myenteric neurons. Detail of a myenteric plexus ganglion in a cross-section obtained from control **(A)** and from a DNBS-treated **(B)** rat colon**.** Hematoxylin-eosin staining highlights prominent distortions in the myenteric plexus ganglion, with nuclear dot-like aggregates in myenteric neurons after DNBS treatment. Arrows show myenteric ganglia (bar 10 μm). **(C-D)** Confocal image of a myenteric ganglion obtained from control **(C)** and **(D)** DNBS-treated LMMPS stained with the neuronal marker HuC/D. After the inflammatory stimulus some neurons displayed a reduced soma and a prominent HuC/D staining in the nucleus (arrowheads). Bar 50 μm. (**E**) Myenteric neuron number normalized per ganglion area calculated in colonic whole-mounts obtained from DNBS-treated (solid bar) and control animals (empty bar). (**F**) Mean myenteric neuron area calculated in colonic whole-mounts obtained from DNBS-treated (solid bar) and control animals (empty bar). Values are expressed as mean±SEM of 4 experiments. **P<0.01 and ***P<0.01 *vs* values obtained in vehicle-treated control animals by Student's t test.
